# Understanding genomic medicine for thoracic aortic disease through the lens of induced pluripotent stem cells

**DOI:** 10.3389/fcvm.2024.1349548

**Published:** 2024-02-19

**Authors:** Aminder A. Singh, Deeti K. Shetty, Aishwarya G. Jacob, Semih Bayraktar, Sanjay Sinha

**Affiliations:** Wellcome-MRC Cambridge Stem Cell Institute, Jeffrey Cheah Biomedical Centre, Cambridge, United Kingdom

**Keywords:** thoracic aortic disease, genetic variants, induced pluripotent stem cells, disease modelling, aortic aneurysm, aortic dissection, 3D models

## Abstract

Thoracic aortic disease (TAD) is often silent until a life-threatening complication occurs. However, genetic information can inform both identification and treatment at an early stage. Indeed, a diagnosis is important for personalised surveillance and intervention plans, as well as cascade screening of family members. Currently, only 20% of heritable TAD patients have a causative mutation identified and, consequently, further advances in genetic coverage are required to define the remaining molecular landscape. The rapid expansion of next generation sequencing technologies is providing a huge resource of genetic data, but a critical issue remains in functionally validating these findings. Induced pluripotent stem cells (iPSCs) are patient-derived, reprogrammed cell lines which allow mechanistic insights, complex modelling of genetic disease and a platform to study aortic genetic variants. This review will address the need for iPSCs as a frontline diagnostic tool to evaluate variants identified by genomic discovery studies and explore their evolving role in biological insight through to drug discovery.

## 1 Introduction

Aortic wall structure reflects its unique function to provide vascular capacitance for every cardiac cycle and predisposes to thoracic aortic disease (TAD) which includes classical pathologies of aneurysm and dissection. These diseases are the end points of molecular failures within the wall, and occur when wall stress exceeds strength resulting in life threatening complications (1). Acute aortic dissection confers high mortality with an estimated incidence of 6 events per 100,000 population (2). Despite the devastating clinical presentation, clinical outcomes for this cohort have seen little improvement over the last 15 years (3). Diagnosis without cross sectional imaging is challenging and is compounded by the lack of a TAD specific biomarker (4). Importantly, there are no pharmaceutical treatments which prevent aneurysm formation or progression effectively, with current focus on reducing biomechanical loading of the aorta through antihypertensives. Patients require regular surveillance imaging and often surgical intervention. Presently, aortic diameter is the main indication for surgical intervention however an acute event can occur with a normal sized aorta. As such, there is need for additional molecular information that can be used in multimodal risk scoring to predict disease severity and determine the need, and timeframe, for intervention.

Rapid development of next generation sequencing technologies has led to genetic testing being increasingly used for Mendelian disease (5) but it remains difficult to functionally validate the huge wealth of data generated. Challenges in assigning causality to genetic variants and studying TAD include high inter- and intra-genic variability in risk and/or type of first aortic event (6), incomplete penetrance, private mutations, and lack of aortic tissue available for mechanistic study. The overarching rationale for genetic testing is critical because individuals can be identified as at-risk for TAD and associated mortality and morbidity are preventable, including for potentially at-risk family. Despite extensive genomic coverage in patients recruited through large databases, such as The 100,000 Genomes Project, many individuals are undiagnosed and uncertainty around assigning causality to variants remains (7), limiting clinical utility.

**Figure 1:**
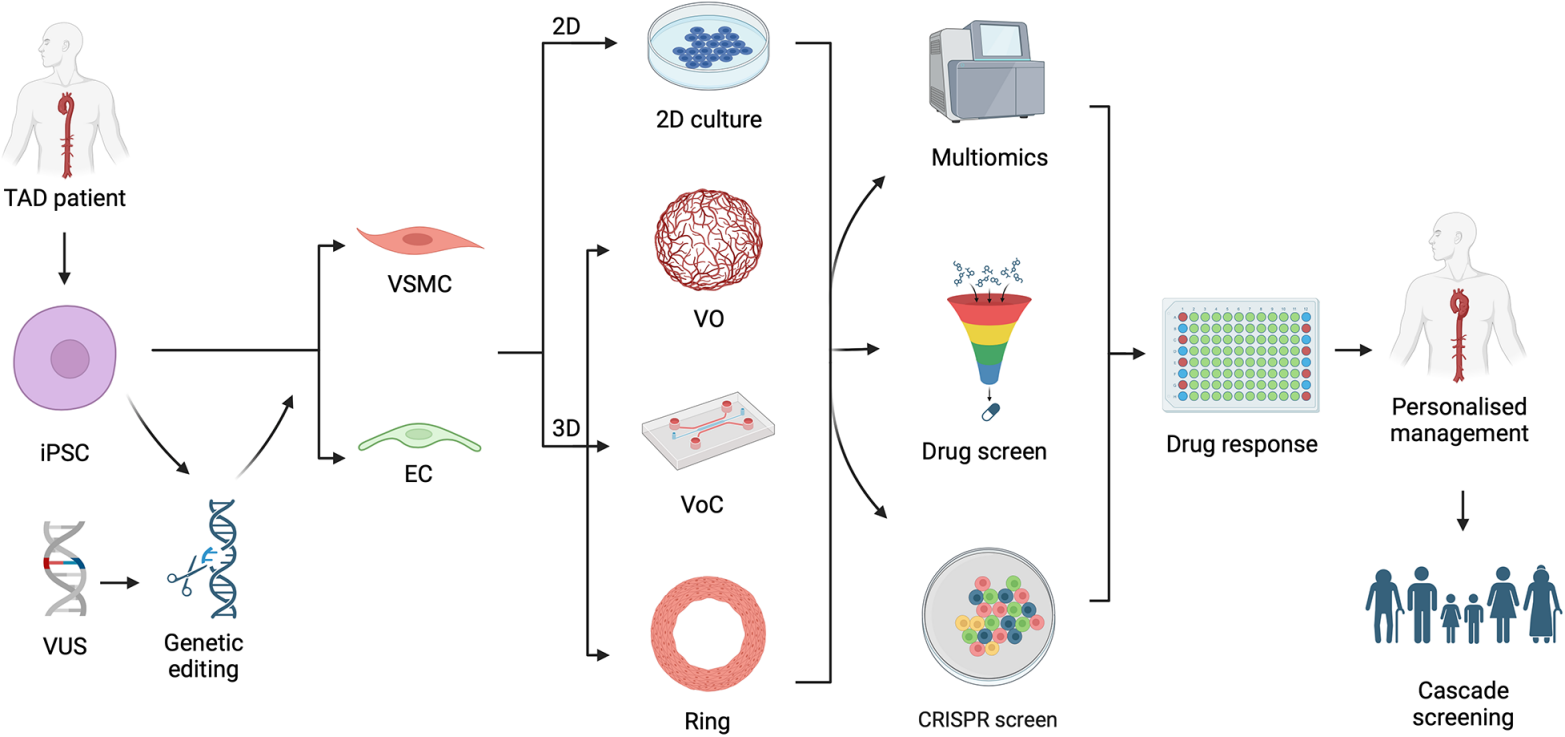
Graphical summary demonstrating the utility of induced pluripotent stem cells (iPSC) in understanding thoracic aortic disease (TAD) variants of unknown significance (VUS). iPSCs can be generated from a TAD patient or an established iPSC line genetically edited to carry a VUS. These can be differentiated in to vascular smooth muscle cell (VSMC) or endothelial cell (EC) in the form of a 2-dimensional (2D) culture or 3-dimensional (3D) culture system including vascular organoids (VO), vessel on a chip (VoC) and vascular ring. These models can proceed to multiomic evaluation, be used for drug or CRISPR screening, and then followed by assessment of drug response in high throughput manner. Such an approach allows for personalised management of the TAD patient and allow determination for need of cascade screening of family members. Figure created with BioRender.com. Publication licence granted by BioRender. Agreement number UH266AGKA0.

Induced pluripotent stem cells (iPSCs) are patient-derived, reprogrammed cell lines which allow mechanistic insights and the ability to create complex models of genetic disease. This review will begin by defining the genetic basis of TAD, key signalling pathways implicated in pathogenesis and discuss unifying theories to aid targeting of therapeutics. Particular focus is given to the latest advances in the use of iPSCs and genetic engineering which can be applied to studying aortic disease variants identified by next generation sequencing (Figure 1). These recent strategies allow for increased mechanistic exploration with a view to expanding personalised management in TAD.

## 2 Vascular abnormalities in thoracic aortic disease

The aortic wall consists of three layers—tunica intima, tunica media and tunica adventitia. The intimal layer, on the luminal surface, is composed of endothelial cells and the basement membrane, separated from the tunica media with an elastic lamina. The tunica media is composed predominantly of concentrically arranged vascular smooth muscle cells (VSMCs), elastin and collagen. The adventitial layer is comprised of fibroblasts, pericytes, immune cells, nerves and lymphatics (8). Key to normal function is the high level of compliance within the aortic wall (9). Increased stiffness results in wall weakness and elevated cardiac afterload resulting in higher cardiovascular events (10) as well as increasing risk for type A aortic dissection when combined with high wall stress (11).

### 2.1 Vascular smooth muscle cell contractility required for vessel wall homeostasis

VSMCs are the most abundant cell type within the aortic wall with a substantial body of evidence, particularly from human genetic data, implicating them in pathogenesis of TAD (12–14). Similar abnormalities in their function and surrounding extracellular matrix (ECM) are observed in both syndromic and non-syndromic TAD, making them a strong candidate for mechanistic research and therapeutic strategy building. Specifically, loss of VSMC contractility is a common feature observed in many hereditary TADs (15, 16). Mutations in VSMC contractile genes are predicted to decrease the cells ability to generate the force needed to regulate the mechanical state of the wall (1). Altered force generation is one of the causes of TADs (17) with mutations in VSMC contractile protein coding genes *ACTA2*, *MYH11* and *MYLK* responsible (18–20). Gain of function mutations in *PRKG1* result in reduction of VSMC relaxation and predisposes to TAD, further implicating VSMC specific genes in pathogenesis (21). As such, contractility loss serves as a good readout in phenotypic assays (22).

### 2.2 Maladaptive extracellular matrix remodelling at the core of aortic tissue failure

Most major vessel groups have a characteristic ECM composition based on function and the aorta is typically is composed of elastin, glycoproteins, collagen and proteoglycans (23). The combination of elastic fibres and fibrillar collagens provides mechanical integrity as well as compliance. Indeed, pathogenic variants in ECM related genes such as *LOX,* encoding for an enzyme which crosslinks collagen and elastin, are causal in heritable TAD (24). There is constant remodelling of ECM in normal physiology through interplay between degrading proteins and their tissue inhibitors, in which VSMCs have a central role through a mechanotransductive feedback loop in response to mechanical load. VSMCs attach to the ECM through focal adhesions and integrins forming the elastin-contractile unit which is involved in force generation and mechanotransduction (25). A more plastic matrix would encourage the VSMCs to secrete fibrillar ECM components, such as collagens and fibrilins, to enable fibre formation. Mechano-adaptive remodelling is a well-orchestrated process entailing transcriptional, translational, and secretory pathway modulation required for normal functioning of the vessel wall (26–30). However, derangement of these processes results in maladaptive remodelling and an abnormal ECM predisposes to TAD. Ultimately, these changes impact cellular processes such as proliferation, cell migration, and contractility. Given this, understanding the VSMC-mediated ECM remodelling and turnover is critical in uncovering of TAD pathogenesis.

VSMC contractile machinery, ECM and TGF-β pathways together are thought to regulate mechanosensing (mechanical stress and strain sensing) by VSMCs and control overall aortic wall health (31). The central hypothesis is that if the sensing of these mechanical cues is abnormal then inappropriate remodelling predisposes to wall failure. Recent mouse studies have highlighted the need for having a mechanoprotective role of *YAP* and *TAZ* for homeostatic functioning of aorta whereby their depletion leads to aortic aneurysm (32). Having fewer functioning elastin-contractile units precludes appropriate mechanosensation and application of VSMC contractility, rendering the aortic wall susceptible to damage at high pressures (1, 33). Aside from contractile unit loss, failure of the structure leads to abnormal focal adhesion complexes and inappropriate sensing of the ECM by the VSMC (34–36).

While diseased human and mouse aortic tissue provides an endpoint view of compromised mechanical integrity, there is limited scope to study matrix dynamics in detail. Use of decellularized ECM for label-free quantification of ECM proteins and glycoproteins using MaxQuant mass spectrometry has been used to study iPSC derived VSMCs in arterial diseases (37). Individual proteins such as elastin and collagen deposition have been evaluated using human VSMCs (38, 39). Secretome forms a huge part of VSMC matrix modulatory phenotypic responses and number of studies have explored this using ELISA-based MMP assays and zymography for MMPs and tissue inhibitors of metalloproteinases (40, 41). The potential of quantifying nascent ECM secretion through BONCAT mass spectroscopy has been shown with other disease models but could be also applied to TAD (42). When it comes to studying the mechanics of VSMCs, measuring both the stiffness of cells and its secreted ECM is key, particularly in 3D models. By using tools such as Atomic force microscopy, uniaxial stress and strain measurements, temporal insights to how elasticity of substrates can influence VSMC cell migration and proliferation can be gained (43, 44). Evidence of ECM stiffness and strain influencing the phenotypic switching of VSMCs was obtained by culturing primary VSMCs *in vitro* (33, 45). As such, TAD patient iPSC VSMCs can also be evaluated for the tendency or potential for phenotypic switching and calcification (46–48).

### 2.3 Controversial role of TGF-β signalling in aortopathies

In VSMC homeostasis regulation, transforming growth factor-β (TGF-β) signalling has a key role in cellular processes of proliferation, differentiation, and ECM remodelling (6). However, there remains considerable debate around whether TGF-β signalling is implicated in TAD or if it confers a protective effect. TGF-β is a VSMC differentiating factor and increases expression of smooth muscle actin, smooth muscle myosin, calponin and myocardin. Contributions of both canonical and non-canonical signalling are integral to embryonic VSMC lineage specification during development and VSMC cell fate (49).

Impaired TGF-β signalling can lead to a loss of VSMC contractility markers and gain of proliferative markers indicating a synthetic phenotype switch. As a result, degradation of ECM and abnormal aortic wall remodelling is seen (50). In Marfan Syndrome (MFS), increased activation of TGF-β is related in part to abnormal Fibrillin-1, which normally sequesters latent TGF-β within the ECM (51). In MFS mouse models, increased TGF-β signalling has been demonstrated (52) with a TGF-β neutralising antibody or an angiotensin II type 1 receptor blocker reversing the disease phenotype (53). However, the exact role of TGF-β is not fully understood with several studies demonstrating a protective role (54–56). Use of TGF-β neutralising antibody conversely resulted in a severe manifestation of aortic disease (57) although the timing of this appears important with a protective benefit demonstrated at a later stage (54). Loeys-Dietz Syndrome (LDS) is defined by mutations in the TGF-β receptor or SMAD2/3 where patients exhibit aortopathy and cardiovascular events are the leading cause of death. In LDS, despite predominantly loss of function mutations in the TGF-β pathway, a paradoxical increase in phosphorylated Smad2/Smad3 complex has been shown in the aortic wall (58, 59) suggesting that even with an abnormal TGF-β receptor, overactivity of TGF-β is implicated in disease. Subsequent studies have identified lineage-specific TGF-β events in distinct VSMC subsets as an explanation for this paradox (60, 61). Nevertheless, it appears the exact role of TGF-β in TAD pathogenesis remains controversial, compounded by its temporal role in multiple cellular processes and crosstalk with other implicated signalling pathways. As such, this is an area where future scientific endeavours should continue to focus.

### 2.4 Intersection of “unifying theories” lead to convergence of key druggable candidates from shared pathways

Given the pathological and morphological similarities between many distinct genetic and polygenic types of TAD, is there a unifying theory for a “final common pathway” leading to aneurysm or dissection? This argues that regardless of the underlying signalling pathway that is perturbed, the changes that result are common across TAD. This includes phenotypic switching of a subset of VSMCs to form fibroblast-like, macrophage-like or adipocyte-like cells, marked by progressive loss in contractility, increased apoptosis, abnormal ECM remodelling alongside inflammation and endothelial cell related oxidative stress (62). These changes are aligned with dysfunctional VSMC mechanosensing which further exacerbates loss of ECM homeostasis through abnormal remodelling (63). Such a theory is attractive as it suggests that regardless of the initiator, therapeutic development could focus on the shared pathways.

### 2.5 Other cell types important in vessel wall functioning and integrity

Although VSMCs appear the dominant cell type in disease, there is evidence of the role of other cell types in the aortic wall. Endothelial cells sense shear stress and crosstalk with VSMCs (64) and endothelial dysfunction has been demonstrated to contribute to aortic wall pathology (13, 65, 66). Endothelial cell function is impacted by dysfunctional ECM through altered distribution of junctional proteins, like occludin and claudins, which has consequence on blood vessel integrity (67). The adventitial layer has a role in remodelling the aortic wall in response to mechanical stress (68). In such conditions, fibroblasts undergo phenotype switching to myofibroblasts which deposit collagen and invoke an inflammatory response (69). Abnormal activation of this pathway contributes to abnormal ECM remodelling and can lead to reduced integrity. Endothelial cells, and particularly adventitial cells, are relatively understudied in comparison to VSMCs in TAD pathogenesis and an approach which encompasses all these cell types is preferable.

### 2.6 Regional aortic heterogeneity

The thoracic aorta can be classified based on embryological origin with the aortic root derived from lateral plate mesoderm, the ascending and aortic arch from neural crest and the descending and abdominal aorta from paraxial mesoderm (70). Abdominal aortic aneurysms (AAA) are associated with hypertension, smoking, and atherosclerosis and are rarely seen as part of a multisystem connective tissue disorder. Aneurysm in the abdominal segment is more common than in the thoracic but the underlying aetiology of these are divergent despite sharing some common abnormalities in VSMC apoptosis, ECM degradation and immune infiltration. Evolving evidence from single cell RNA sequencing demonstrates both molecular similarities and differences between abdominal and thoracic aortic aneurysm (71). Despite this, genetic abnormalities play a significantly less important role in aneurysmal degeneration of the abdominal vs. the thoracic segment, in particular there is no clear inheritance pattern in AAA. Recent endeavours have focussed on collating AAA implicated genes, such as in the AAAKB, to aid genetic study (72). No published iPSC models focus on AAA but VSMCs of paraxial mesoderm lineage (73) could be generated with view to establishing it. As such, this review will retain focus on TAD.

Regional heterogeneity within the thoracic aorta is thought to be due to differential lineage specific response due to diverse embryological origin on the constituent cells. A recent study of single cell sequencing of lineage traced secondary heart field (SHF) derived VSMCs from the aortic root of Marfan mice (C1039G) revealed a distinct transcriptomic profile from the neural crest derived aortic SMCs and the activation of specific pathways such as integrin α5 signalling (74). That combined with the preponderance of MFS aneurysms in the aortic root, imply that secondary heart field derived VSMCs are a critical cell type to be examined. Gong et al. (61) demonstrated abnormal SMC contractility and ECM deposition in lateral mesoderm derived VSMCs suggesting a lineage specific response to a *SMAD3* variant. This differential response can be evaluated using specific iPSC differentiation protocols producing VSMCs from lateral mesoderm, neural crest, and paraxial mesoderm (73, 75, 76). Such an approach can provide mechanistic insight into disease distribution in the aorta given that LDS has higher propensity of aortic root aneurysm, where there is predominantly lateral mesoderm derived VSMCs (60), and can aid clinicians in assigning region-based size thresholds for intervention.

## 3 Disease modelling of TAD

The difficulty in obtaining early-stage tissues from patients with TAD and the challenges of clinical trials mean that we need to use suitable model systems to understand pathogenicity and identify and test novel therapies. The complexity of the aortic environment, with multiple cell types featuring complex inter- and intra-cellular signalling, extensive vessel wall and vascular niche remodelling along with varying haemodynamic loads in a three-dimensional space makes this a challenging endeavour. Nevertheless, we would do well to remember the observation by George Box that “All models are wrong but some are useful” (77). Consequently, we will briefly review common *in vivo* models of TAD before describing iPSC based *in vitro* systems in some depth and discuss their advantages and disadvantages.

### 3.1 *In vivo* models offer a window into studying spatial interactions but are restrictive in mimicking human disease complexity

While *in vivo* modelling using genetically engineered rodents offers an important solution for the spatial and systemic complexity of vascular diseases, they remain somewhat limited in their scope. The pathways affected in these models are not necessarily conserved with human disease. In monogenic aneurysmal disorders such as MFS and LDS, some mutant mouse models have captured aneurysm formation and dissections and have been useful in delineating the impact of these mutations in disease progression. However, these models are low throughput and capture only a small proportion of the mutational landscape seen in humans. For example, MFS has been reported to be associated with >1,500 mutations in *FBN1* featuring missense mutations and splicing errors that lead to truncations (78). The two most widely used mouse models of MFS are the C1039G (79) and the MgR (80) which contain a missense and hypomorphic mutation in the *FBN1* gene respectively. Further, these inbred models do not capture the heterogeneity arising from the genetic background of the affected human population and are not fully effective at predicting drug responses, a classic example being that of losartan that proved remarkably effective *in vivo* in the C1039G/+ model MFS (53) but did not perform as well in clinical trials (81).

Therefore, to accurately model human genetic diseases it appears that a humanised model may have some advantages based around the conserved genetic background. *In vitro* models also have a wider scope than *in vivo* models for high throughput studies and allow detailed molecular characterisation, phenotype assessment, mechanistic exploration, and drug testing. *In vitro* models using primary vascular cells—smooth muscle or pericyte and endothelial—can be highly technically challenging. For example, VSMCs are phenotypically plastic and tend to lose their contractile properties and alter their molecular profiles once dissociated from tissue and cultured. Moreover, primary cultures display considerable variability making reproducibility a major challenge.

### 3.2 iPSCs based modelling addresses genetic diversity of the mutation landscape

The advent of iPSCs has been a game changer for disease modelling using direct patient-derived culture systems. They provide an ethical source of cells and disease models compared to *in vivo* systems, and even embryonic stem cell-based platforms. Typically, skin fibroblasts or peripheral blood mononuclear cells are collected from the patient and re-programmed directly to produce pluripotent stem cells that retain the genetics of the somatic cells they were derived from (82–86). iPSCs can then be efficiently differentiated into a variety of cell types, and in the context of disease these iPSC-derived cells can be used as effective patient-specific models (Table 1). This makes them an incredibly versatile and high-throughput platform for modelling disease states and capturing the heterogeneity between patients. Attempts to use iPSCs to model vascular cell types began in the late 2000s (93, 94) resulting in a mix of several cells including VSMCs and endothelial cells (ECs), in a form of co-culture. Since then, a large number of protocols have been described using highly defined media with small molecules or cytokine cocktails to drive differentiation [several of which are well summarised in these reviews (95–97)] that claim to have successfully produced VSMCs or ECs of high purities, organ-specific, developmentally pertinent lineages (73, 76) and fine-tuned phenotypic states including the contractile one (37, 98, 99).

**Table 1:** iPSC models of thoracic aortic disease that have been phenotypically characterized and reported.

Disease	Mutation within iPSC	Phenotypic characterisation	Rescue of phenotypic defect
Marfan Syndrome (87)	FBN1 exon 30 (c.3725G > A) FBN1 exon 21 (c.2638G > A)	Comparison of mutant and isogenic VSMCs FBN1 deposition, MMP expression, Cell contractility and death. Cell stretching aggravates the phenotype.	Yes. Inhibition of p38-MAPK or KLF4 knockdown
Marfan Syndrome (40)	FBN1 exon 30 (c.3725G > A) FBN1 exon 14 (c.1837 + 5G > C) FBN1 exon 10 (c.1051C > T)	Comparison of mutant and isogenic VSMCs MMP expression by DQ gelatin and activity through Zymography, Cell death and cell proliferation	Yes. GSK3b inhibition reduces proteolysis by MMP secretion and apoptosis
Loeys-Dietz Syndrome (88)	TGFBR1 c.688G > A	VSMC lineage-specific effects. CPC derived VSMCs have disrupted canonical TGF-β signalling and decreased gene expression of VSMC markers and contractility markers. Abnormal ECM formation.	Yes. Combination treatment with activin A and rapamycin that could rescue the SMC defects caused by the TGFBR1 variant
Loeys-Dietz Syndrome (61)	SMAD3 c.652delA	SMAD3 mutant VSMC 3D-vascular ring model had increased stiffness	Inhibition of mir-29 partially rescued ELN expression in SMAD3 variant
Loeys-Dietz Syndrome (89)	TGFB3 p.Asp263His	Reduced contractility of neural crest VSMC upon thromboxane A2 stimulation. Variability between clones observed	No
Bicuspid aortic valve- (BAV-) related thoracic aortic aneurysm (90)	Notch1 mutation not found but no other mutation was specified	Lineage specific effect of neural crest-derived VSMC showing reduction in VSMC marker and contractility marker expression. TGF-β signalling was decreased. Paraxial mesoderm derived VSMCs were not affected in these aspects	Yes. Inhibition of mTOR pathway using rapamycin rescued defects
Williams-Beuren syndrome (WBS) and Supra Valvular Aortic Stenosis (91)	Hemizygous microdeletion of a 1.5–1.8 Mb region on 7q11.23 involving 26–28 genes, including ELN	Immature VSMC phenotype having reduced levels of elastin and differentiated SMC markers Functional immaturity due to lack of response to Carbachol stimulation for calcium transient generation	Yes. EBPL2 is a synthetic peptide that contains a sequence, which is homologous to the human elastin domain. EBPL2 binds to elastin-binding protein (EBP) to stimulate activity and induces elastin receptor complex-dependent signaling
Supra Valvular Aortic Stenosis (92)	4-bp nucleotide (GTAT) insertion in exon 9 of ELN that was predicted to result in a frameshift and a premature termination codon in exon 10; 2 clones were characterized	VSMCs have defective actin filament bundle formation Hyperproliferation and migration of VSMCs is observed Increased ERK1/2 activity is implicated in hyperproliferation of SVAS iPSC-SMCs	Yes. Elastin and Small GTPase RhoA rescue phenotype defects

### 3.3 Single cell transcriptomic studies have hailed the in-depth study and comparison of *in vitro* iPSC-derived cell types and *in vivo* counterparts

The rapid expansion of single cell transcriptomics has facilitated nuanced descriptions of individual vascular cells in different tissues and their developmental trajectories which, in turn, will pave the way for further advancements toward highly precise and effective differentiation protocols to achieve the exact cell state needed to model a specific condition. The ability of these protocols to produce vascular bed or niche-specific cells is important because some vascular diseases often show region-specific susceptibility to aneurysm formation e.g., aortic root aneurysms in LDS. Our own studies on MFS have provided huge insights into how patient iPSC-derived VSMCs are capable of clearly reflecting disease severity *in vitro* while uncovering the differential response to drugs between patients (22, 40) and hence are an invaluable platform for the development of personalised, tailored therapeutics.

Despite these advances in iPSC-based modelling of patient-specific disease, there are several problems associated with these systems. Personal insights on technical aspects and limitations of iPSC models of aortic disease have been extensively discussed previously (22). Heterogeneity can be a major issue when trying to decipher pathways commonly perturbed in patients. While this can be a strength in modelling varying genetic backgrounds, particularly for disease susceptibility and drug responses, it can pose several technical challenges that might undermine the findings from such models. Another problem is modelling the correct cell type i.e., the appropriate source—vascular bed, lineage and/or regional specification on the vessel with respect to hydrodynamic forces. Directed differentiation protocols now allow for specification of several of these features in VSMCs and ECs. The incorporation of specific chemical cues, mechanical strain and flow-induced shear stress have helped to further fine-tune cell phenotypic state e.g., contractile or synthetic or phenotypically switched. Work to increase the maturity of iPSC derived cells is ongoing with modification of differentiation protocols based on sequencing data. However, these disadvantages are far outweighed by the benefits of iPSC-based disease modelling and therapeutic targeting.

## 4 Genetic modulation for mechanistic insight

Utilising tools to manipulate cellular behaviour has advanced mechanistic understanding of disease. Particularly, DNA-level modulation offers stable and inheritable alterations, a vital tool in foundational research and therapeutic development, and has been useful in investigating cardiovascular disease. Progress in genome editing has augmented our toolkit, providing the ability to enact DNA modifications through numerous strategies across diverse frameworks, enabling either site-specific or random edits, and classifications based on modification nature (e.g., knock-ins, knock-outs) or timeliness (e.g., temporal, constitutive). Site specific edits allow manipulation of genetic information at endogenous genes despite presenting considerable challenges, such as hard-to-identify off-target effects. Moreover, technologies such as the Tet ON/Tet OFF systems allow temporal control over genetic modifications and their subsequent cellular impacts, thus widening the scope and potential applications of genome editing technologies (100, 101).

### 4.1 CRISPR systems for gene editing

The conventional CRISPR approach hinges on the Cas protein inducing a double-stranded break (DSB) in the DNA, where the guide RNA finds complementarity in the genome. Subsequent repair of this intentional DSB is executed via the cell's intrinsic replication machinery, chiefly through two primary pathways: Non-Homologous End Joining (NHEJ) and Homology-Directed Repair (HDR) (102, 103). The evolution of CRISPR-Cas technology also brought the emergence of diverse Cas proteins, base editors, and prime editors which contribute to enhanced specificity and versatility of genome editing techniques. Engineered variants of Cas proteins have been pivotal in reducing off-target editing (104, 105) and enabling access to a broader genomic region, thereby expanding the scope of potentially editable regions (106). Moreover, the development of base editors, which use catalytically repressed or inactive Cas9 fused to a DNA deaminase enzyme, provide a means to edit single base pairs without generating a double-stranded break (DSB) (107). Using base editing, a recent study investigated the causal variants implicated in blood pressure through VSMCs (108).

A more recent approach, prime editing, utilizes a Cas9 nickase fused to a reverse transcriptase, alongside pegRNA which is produced through the fusion of the repair template with the guide RNA (109). pegRNA therefore serves as a template for the reverse transcriptase to synthesize the edited strand, which then integrates directly into the genomic DNA, while guiding the Cas9 to the target site, collectively minimizing indel formation and off-target effects. While these newer technologies hold their own limitations, such as the inability to generate transversion mutations and potential inadvertent RNA edits with base editing, as well as the restriction on sizable insertions with prime editing, continuous improvements in methodologies have expanded the efficiency and toolkit of prime editing, propelling forward the evolution of CRISPR technology.

Modulating cellular behaviour extends beyond manipulations at the DNA level, encompassing various regulatory layers, including the epigenome and RNA. The CRISPR domain has been adeptly utilized for such purposes, especially through innovative strategies involving engineered proteins. For example, by coupling CRISPR with a catalytically deactivated Cas protein and subsequently fusing it with specific domain allows modulating epigenetic behaviour (110), adding a nuanced level of genetic control without altering the underlying DNA sequence. Additionally, different Cas proteins can be employed to orchestrate edits at the RNA level, providing a mechanism to regulate gene expression post-transcriptionally (111).

### 4.2 Genetic modifications in iPSCs for disease modelling

iPSCs themselves are amenable to genetic modulations including via CRISPR-Cas9, making them a powerful tool to characterise the mechanisms underlying disease aetiology. For instance, “CRISPR-correction” of patient iPSCs at mutated loci into wildtype sequences, allows for the generation of isogenic iPSC lines that preserve the genetic background of the patient while establishing causality of the mutation in question (87). What this also means is that patient-specific differences in disease susceptibility due to genetic background, for example the effect of accompanying SNPs, can be modelled in the absence of the confounding “main” mutation. Several studies have used iPSCs and derived vascular cells—VSMCs, pericytes and ECs—to model a variety of vascular diseases including, pulmonary arterial hypertension (112–119), Hutchison-Gilford progeria (120–124), atherosclerosis (47, 125, 126), neurodegenerative cerebro-vascular conditions like Moyamoya disease (127–131), CADASIL (132–135), diabetes and its associated conditions (136–138).

Coupling CRISPR gene editing technology with iPSCs has allowed for significant enhancement of aortic disease models such as syndromic aortopathies (22, 40, 87, 88) and aortopathy genetic variants (61, 139). Correction of a *FBN1* mutation demonstrated rescue of *in vitro* MFS disease phenotype (87). iPSCs can determine causality of a disease variant of interest (61, 140) and provide information for precision medicine and need for cascade screening. Further, researchers have generated reporter lines of *ACTA2* and *MYH11*, which are used in improving stem cell derived VSMC differentiations (99).

Despite challenges, each advancement in the CRISPR technology has gradually improved the specificity and applicability of these vital tools, enabling unprecedented insights into genetic mechanisms. This has facilitated innovative developments across diverse biological domains, with potential applications in many more non-syndromic aortic diseases.

## 5 Genetic abnormalities in thoracic aortic disease

In heritable TAD, irrespective of penetrance, 25% of patients have at least one first degree member affected (141, 142). Patients with a family history of TAD demonstrate more rapid aneurysmal growth than sporadic TAD or MFS aneurysm (31). Interestingly, there is significant variation in aortic event risk, even within functionally related genes, such as those regulating VSMC contraction (6). Such genetic information has been used to predict those at risk of severe, early onset TAD and those with late onset low penetrant TAD (6, 143).

Initially highly penetrant monogenic variants were identified with a candidate gene approach and linkage analysis. More recently, next generation sequencing has accelerated novel variant identification but poses some challenges. Firstly, it can be unclear whether the variant is causal, particularly if it is within a gene not previously associated with the condition. Judging causality of a genetic variant based on its presence within a known disease related gene is an inherently biased approach and could hinder discovery of new pathways. Identifying variants of unknown significance (VUS) poses a challenge to clinicians (144) and can make genetic counselling challenging and raise uncertainty in need for cascade screening. Additionally, penetrance classification and determining pathogenesis are critical in design of pharmacological therapy or determining prophylactic treatment. Despite this, the correlation between genotype and phenotype in most TAD cases remains poor and additional techniques to predict clinical course in patients are urgently required.

### 5.1 Novel variants with lower penetrance identified by next generation sequencing can be assessed for causality using iPSC-based disease modelling

iPSCs were first used to model genetic variants which result in substantial modification of a protein product, but this technology can be used in more advanced models where complex traits are investigated. Many genetic variants identified are within intronic or intergenic regions and can regulate gene function (145). Variable expressivity of monogenic disorders may be due to modulation by additional genetic variants and incomplete penetrance. A genome-wide association study (GWAS) of TAD in the Million Veteran Program revealed 21 risk loci with suggestion that TAD is a distinct vascular disease with complex traits. Importantly, this work argues that TAD is not inherited exclusively through protein altering variants of large effect size but are due to a combination of multiple at-risk genetic variants (146). Similarly, GWAS of the UK Biobank revealed 14 overlapping loci in ascending and descending thoracic aortic aneurysm (147), adding to findings from earlier GWAS (148–150). Given these interesting findings, the need for functional validation and exploration of biology underpinning disease is clear. Diseases which encompass a combination of numerous low effect risk loci, often identified by GWAS, pose a challenge in an iPSC model. Each individual variant may be too subtle to demonstrate a phenotype *in vitro,* which is in part due to gene-gene interactions not present outside of the patient's genetic context (151). However, patient derived iPSCs, which carry all potential risk variants, can be used to demonstrate which regions are more relevant in phenotypic manifestation. Multiple patient derived iPSCs can used in a pooled culture creating a “cell village” where the impact of variation can be studied at a population scale, providing in depth information of the effect of expression quantitative trait loci (eQLT) (152). The impact of eQLT on phenotypic manifestation of a genetic variant can be addressed using patient specific iPSCs and then CRISPR correcting these regions to create isogenic controls (153). As such, risk loci can be evaluated to assess their functional impact on common genetic variants which in turn can feed into individualised risk scoring.

### 5.2 Use of iPSCs for determining the pathogenicity of novel genetic variants

Heritable TAD is non syndromic and typically associated with a single mutation in a gene related to ECM, TGF-β signalling or VSMC contraction (154). Over time an increasing number of variants are being reported and genes classified as pathogenic (155). Substantial evidence exists for *ACTA2, MYH11, MYLK, LOX* and *PRKG1* but other genes are implicated with varying levels of certainty (155). Most of the VUS are not causal but may be low penetrant risk variants contributing to TAD (143). Even in large, national whole genome sequencing studies the diagnosis rate in heritable TAD remains low (7). There is consequently great potential for using iPSC models from heritable TAD patients or by generating CRISPR knock ins in the hope of determining causality and providing clinicians with more biological information to inform future management plans.

Mutations in *FBN1* are associated with MFS which is the commonest single gene aortopathy. Currently there are over 3,000 *FBN1* pathogenic variants identified with most unique to a family (156). Genetic studies have demonstrated that cardiovascular risk is determined by the genetic variant possessed. Those with haploinsufficiency *FBN1* variants have increased risk of major cardiovascular events than dominant negative mutations (157). Within the dominant negative group, variants which effect or create Cysteine residues and in frame deletions in exons 25–36 and 43–49 have over a 6-fold increase aortic event risk (156). Despite this, delineating the genotype-phenotype relationship, particularly in MFS, remains a major challenge. Genetic background is important as presence of another TAD associated variant can predispose to a more severe aortic disease phenotype (158). Genetic background can also partly explain the difference in phenotype severity within a family who carry the same *FBN1* mutation (22) but there are other factors at play which remain uncovered. An iPSC model could be used to provide an *in vitro* prediction of severity once appropriate parameters of assays have been defined. Such an approach would be particularly useful in a young patient with a novel *FBN1* variant where the impact of intensive lifelong surveillance is significant. Similarly, each LDS subtype carries different severity and therefore the relationship between genotype and phenotype is important. iPSC models of LDS have demonstrated abnormal contraction and enhanced proliferation of VSMCs (88) and can be utilised to explore genotype-phenotype correlation. As new variants are discovered in LDS related genes, there is an emerging need for gene-based classification and personalised risk scoring for each patient (159).

## 6 Complex three-dimensional disease models

The vasculature is a complex organ that involves crosstalk between the cells of the various layers and their extra-cellular micro-environment. In 2D systems, even in co-cultures of multiple cell types, the organisation of a vessel wall or physiological cell-cell contact cannot be recapitulated. In 2D systems, cells are cultured on hard plastic surfaces whose stiffness far surpasses that of a physiological matrix which can promote a more synthetic VSMC phenotype. Further, iPSC derived VSMCs are not as mature as those derived from tissue which is partly due to a lack of pulsatile flow and mechanical force in a monolayer culture. Hence, it is necessary to model the three-dimensional, multicellular vessel wall anatomy using cells embedded in an appropriate extra-cellular scaffold to capture the full phenotypic manifestation of a healthy or a diseased vessel. When comparing uniaxial stretch in 2D culture to a 3D construct, there is significantly higher increase in VSMC specific markers *ACTA2* and calponin (160) suggesting that a 3D environment allows for more robust cell-cell and cell-matrix interactions. For this purpose, several 3D models (161) have been in development over the last several years that incorporate multiple cell types in a bio-degradable matrix. Some key formats include vascular organoids (VOs) (138), vessel on a chip microfluidic device (VoC) and bioink-based extrusion or bioprinting. Use of these 3D models is on the rise in conjunction with iPSC-derived vascular cells to understand how disease affects vasculature including formation or structure or integrity.

### 6.1 Vascular organoids mimic tissue niches with functional cell-cell and cell-ECM interactions

VOs can be made as simple, self-assembling structures with cells cultured in a hanging drop or in low adherence culture substrata. These are often spherical and in co-cultures of VSMCs and ECs, there is a level of self-organisation between the cell types. Co-culturing VSMCs and endothelial cells has been shown to improve SMC differentiation (162). However, these models tend to have high variability. To obtain a more controlled structure, scaffolds using extra-cellular matrix like collagen, Matrigel or other biomaterials are used and depending on the design, these can result in self-assembly of intricate branched vessel networks with a perfusable lumen lined by ECs and VSMCs or mural cells. VOs can be transplanted and engraft with host tissue to form vascular trees (163). A recent study used human iPSC-derived VOs to study basement membrane thickening seen in the skin microvasculature of diabetic patients which demonstrated vasculopathy. With exposure to high-glucose media, the VOs showed increased collagen IV production and captured the basement membrane phenotype (137). However, similar treatments of 2D iPSC-derived ECs did not show a similar result suggesting the usefulness of 3D models in capturing disease specific phenotypes.

### 6.2 Vessel on a chip enables shear stress, flow and associated mechanotransduction responses

VoCs on the other hand are specifically designed microfluidic devices that can then be lined with ECs, VSMCs and other cells to induce network and vessel formation (164). This model has been well characterised recently with robust vascular network formation comparing primary and iPSC-derived vascular cells, namely ECs, VSMCs and mural cells. The group also established the functionality of these vascular networks—agonist mediated stimulation of VSMCs—and crosstalk between VSMCs and ECs mimicking the environment in a vessel wall (165, 166) validating the usefulness of this system to model vascular diseases where such architecture and cell-cell interactions might be disrupted. Adding flow into a 3D culture system allows endothelial cells to detect shear stress and with this cue they can modulate SMC differentiation through signalling pathways such as nitric oxide (167).

Bio-printing uses cells mixed into collagen or other matrix materials as a bio-ink to be extruded into moulds where vessel formation would be observed (168–170). Numerous assays can be performed including transcriptomic and proteomic analysis, alongside classical ELISA, immunostaining, and Western blotting (164). Multiple chips can be connected in parallel to create complex models more reminiscent of human physiology (171). iPSC derived VSMCs from a patient with Hutchinson-Gilford Progeria syndrome in a chip system showed elevated DNA damage and inflammation, exacerbated by mechanical strain (124). An “aorta on a chip” approach in congenital aortic valve disease demonstrated impaired mitochondrial function due to suppression of *NOTCH1*, reducing VSMC contractility which was partially rescued with drug treatment (172). Liu et al. (173) developed a VoC model with VSMCs of all three aortic lineages in series and determined the differential response to Ciprofloxacin and identified the PI3K-Akt signalling pathway as key in response to stretch (173). It should be noted that these “aorta on a chip” models do not reflect the structure, scale and stress in the aortic wall and so their predictive power over simpler systems is debatable. Nevertheless, VoCs will continue to evolve, and are increasingly useful for their strength of potential downstream processing of cells for further investigation of mechanistic details especially with a systemic-like drug dosage procedure.

### 6.3 Larger vessel-like structures for assessment of structural integrity and tissue failure

Another interesting approach is to use 3D self-assembling tissue engineered vascular rings to provide a readout of aortic wall integrity. Dash et al. (174) fabricated vascular rings using iPSC derived VSMCs and were able to model supravalvular aortic stenosis with uniaxial mechanical testing. This approach is advantageous as it is relatively quick to fabricate the rings once VSMCs have been differentiated. Vascular rings can assess the effect of both mutant VSMCs and ECs in combination, or in isolation, and determine the impact on a clinically relevant readout of burst pressure. However, mechanical loading and integrity in the ring are dependent on the homogenous compaction of cells and deposition of collagen which can frequently be non-uniform leading to point weakness of the ring due to a technical issue (Sinha lab, unpublished). An engineered vascular tissue approach using murine VSMCs to study ECM remodelling allowed for tensile strength testing and assessment of responsive to contraction agonists (175). Here, both protein and proteoglycan accumulation changes over time with vascular calcification changes were demonstrated. While this model is still under development to be used for tensile strength testing of human iPSC VSMCs and its derivative ECM, it does provide a good basis for establishing the same. Use of TAD patient-derived iPSCs VSMCs or fibroblasts can help massively to study transient as well as long-term changes in ECM using 2D and 3D models. Recently, a tissue engineered blood vessel model of Hutchinson-Gilford Progeria Syndrome containing patient iPSC-derived VSMCs and ECs, was shown to serve as a useful drug testing platform where key disease-specific phenotypes such as vascular calcification, VSMC loss and extracellular matrix deposition were clearly captured (120–122).

## 7 Translational potential of iPSCs

Crucially, no drug treatment can halt aneurysmal degeneration of the aorta hence there is urgent need for new therapeutics. Promising animal data for Losartan, an angiotensin receptor blocker, in MFS were not replicated in human trials (176, 177). The AIMS trial with Irbesartan was successful but only showed a very small benefit (0.22 mm/year) in slowing of aortic wall aneurysmal dilatation (178). This was certainly less than seen in the dramatic mouse studies and may reflect differences between mouse and human disease as well as heterogeneity of human populations.

### 7.1 Use of functional genomics to model disease associated with predicted risk loci and severity in iPSC-based screens

Over the years, a multitude of screening methods have emerged as methodologies in functional genomics to offer profound insights into gene functionality and regulatory networks, through both loss-of-function and gain-of-function screens. These range from CRISPR based methods to RNA by employing siRNAs or shRNAs to post-transcriptionally mitigate gene expression. CRISPR activation (CRISPRa) and CRISPR interference (CRISPRi) screens, characterized by systematic overexpression and repression of target genes respectively, offer a platform for identifying genes fundamental to varied cellular activities, such as proliferation and drug resistance (179, 180). Moreover, coupling CRISPR screening with single-cell RNA sequencing delves into the cellular transcriptional outcomes consequent to genomic perturbations, providing an insight into cellular heterogeneity and the varying strategies cells deploy to manage therapeutic interventions (181). These methods have been instrumental in delineating genetic networks, understanding the heterogeneity in cellular responses to genetic perturbations, and unmasking the differential susceptibilities among individual cells within a population. Functional genomics also allows for modelling stroke and other cerebral small vessel diseases where predicted risk loci are validated using iPSC-derived vascular smooth muscle cells and testing for classes of drugs such as HDAC inhibitors and MMP inhibitors (182–184). Recent studies have highlighted the ability to employ iPSCs-derived cells, genomics, and machine learning-based analysis for predicting risk of occurrences of conditions like arrhythmia and heart failure susceptibility in cardiomyocytes (185, 186). Risk loci of coronary artery disease (CAD) (125) and schizophrenia severity (187) were also determined using iPSC-derived VSMCs and neurons respectively. In a recent move, single cell multi-omics that combines transcriptomic and epigenetic data has been used to determine the genetic determinants of arrhythmia (188). Large patient sample collection biobanks are being built across various countries thereby providing ample opportunity to apply functional genomics and build accurate severity prediction models employing the latest tools mentioned above.

### 7.2 Phenotypic screening and drug discovery and in iPSC models offers potential for disease severity prediction

iPSCs can be used to identify deregulated signalling pathways *in vitro* and assess response to compounds hypothesised to improve relevant readouts (113). As such, they serve as excellent drug testing platforms for development of disease-specific therapeutics. For example, pharmacological inhibition of PDGF signalling in iPSC-derived cardiomyocytes in LMNA-related dilated cardiomyopathy, ameliorated the arrhythmic phenotypes (189). Further, even disease severity modelling and prediction both have been successful in (a) Brugada syndrome where cardiomyocytes are affected by arrhythmogenesis of different severity (190) (b) Marfan syndrome where proteolytic degradation by vascular smooth muscle cells was used to scored disease severity and response to drugs inhibiting Gsk3β activity to restore proliferation, curtail apoptosis and MMP secretion (40) and (c) Alport syndrome where kidney organoids modelled severity based on extent of accumulation of Collagens and a marked reduction in the same was demonstrated by the use of chemical chaperones (191). Atherosclerotic plaque stability was demonstrated by KLF4 attenuation by ANGPTL4 treatment of iPSC-derived vascular smooth muscle cells and in mouse models (192). Currently, with very large drug and compound libraries being readily available through pharmaceutical companies, we are only limited by not having widespread use of robotic drug screening platforms and automated phenotype assessment modules (whether imaging or spectrophotometry based) to identify and validate disease modifying therapies.

### 7.3 Drug repurposing and use of omics approaches shorten therapeutics identification time and enhance efficiency

Current technologies of phenotypic drug screening have come far from relying on low throughput 2D cell culture assays along with time-consuming analysis, to high throughput or high content screening assisted by automated analysis (193). In high throughput drug screening, iPSCs can be used to find novel treatments, repurpose drugs or to predict effect (194). Pre-existing drugs for different pathologies are being repurposed for cardiovascular disease based on the phenotypic screening which assays for reducing disease-modifying effects. This kind of approach significantly reduces costs and time incurred in bringing a drug from benchside to clearing clinical trials. Re-engineering existing drugs to enhance efficacy using knowledge of medicinal chemistry, rational drug design to optimize design, delivery and pharmacokinetics in animal models is being done. Such re-engineered drugs are being screened and validated with iPSC models (195, 196). Going an extra mile to determine the complete extent of impact a given drug on modifying global gene expression in a diseased cell has been done in the latest studies by employing single cell transcriptomic profiling of drug response singularly or in combinations using Combi-seq (197). With such advancement we are no longer dependant on gene expression of 1–2 downstream targets to know the efficacy of drug treatment, rather an unbiased and wholistic assessment of direct and indirect target gene changes can be done.

### 7.4 “Clinical trials in a dish” and precision medicine

Drug screening with iPSC-derived cells can enable a precision medicine approach with individualised therapeutic plans based on response in iPSC models and with the knowledge about patient's lifestyle, genetic background, and environment (198). This platform can identify non-responders to drug therapy and in parallel be used to determine the molecular basis for this. Given that iPSCs can identify drug targets, predict response levels, screen novel compounds, and repurpose licensed therapeutics, they provide an excellent resource to bridge the gap between preclinical investigation and clinical trials. “Clinical trials in a dish” can fulfil the requirement of testing various drug therapy parameters in a high throughput fashion. Identifying those at higher risk of experiencing drug related complications can uncover the mechanisms leading to this and tailor therapy to mitigate the risk, such as reduced dosing regimens (199). Another important consideration is the vast resource and human impact of participating in a clinical trial. Using iPSC based “clinical trials in a dish” would complement animal studies and provide important biological information which could confirm the need for, or advise against, human trials. “Clinical trials in a dish” using iPSCs have been used for different cardiovascular cells such as endothelial cell function and crosstalk with cardiomyocytes improvement by Lovastatin in *LMNA* cardiomyopathy (200). Two hERG-blocking drugs, Dofetilide and Moxifloxacin, were also tested on iPSC derived cardiomyocytes for QT prolongation (201). With continued improvement in iPSC differentiations, assay development and 3D modelling, the utility of this approach will only increase.

### 7.5 Cell therapy in aortic disease

A stem cell therapy-based approach has been on keen interest within the cardiovascular field. In aortic context, work has focussed predominantly on AAA with use of mesenchymal stem cells (MSCs) (202), umbilical cord stem cells (203), and iPSC derived smooth muscle cell progenitors (IPSC-SMP) (204) with delivery methods including intravenous injection, direct injection and through collagen scaffolds.

Perivascular delivery of iPSC-SMP, integrated into a collagen scaffold, demonstrated feasibility, VSMC retention and reduced macrophage invasion in a murine model of AAA (204). However, in contrast to the primary VSMC collagen scaffolds, iPSC-SMP scaffolds did not result in reduced aneurysmal expansion. This could in part be due to using smooth muscle cell progenitors as the chosen cell type for delivery instead of more mature VSMCs which would be less likely to dedifferentiate and phenotype switch. An MSC intravenous infusion based clinical trial in patients with small AAA is underway with results awaited (205).

Cell based therapies for aortic disease have shown promise in animal models, but selection and optimisation of the delivered cell type is key. Further work is needed to elucidate whether injection or scaffold-based approaches are more efficacious.

## 8 Conclusion

Coupling the wealth of data from genomic discovery studies with functional validation tools such as iPSCs is key in uncovering further genetic landscape in TAD to aid diagnosis, personalised management and rationalise cascade screening. The versatility of iPSCs and the capability for genetic editing allows for a powerful humanised model for mechanistic insight and risk determination alongside evolving roles in drug discovery and validation. The increasing accuracy of genetic editing and advancement in 3D aortic disease modelling paves the way for patient and mutation specific management approaches and an exciting era of precision medicine in aortic and other cardiovascular diseases.
